# Development of a rapid diagnostic test based on loop-mediated isothermal amplification to identify the most frequent non-typhoidal *Salmonella* serovars from culture

**DOI:** 10.1007/s10096-023-04571-3

**Published:** 2023-02-22

**Authors:** Birgit Edel, Stefan Glöckner, Sylvia Stoll, Nora Lindig, Katharina Boden, Lars Wassill, Sandra Simon, Bettina Löffler, Jürgen Rödel

**Affiliations:** 1 Institute of Medical Microbiology, Jena University Hospital, Friedrich Schiller University of Jena, Jena, Germany; 2 Synlab MVZ Weiden GmbH, MVZ Thuringia, Oncoscreen, Jena, Germany; 3 Amplex Diagnostics GmbH, Gars-Bahnhof, Germany; 4 Division of Enteropathogenic Bacteria and Legionella, National Reference Centre for Salmonella and Other Enteric Bacterial Pathogens, Robert Koch Institute, Wernigerode, Germany

**Keywords:** *Salmonella*, Serovar, Diagnostics, LAMP

## Abstract

Identification of *Salmonella* serovars is performed by conventional seroagglutination or sequencing. These methods are labor-intensive and require technical experience. An easy-to-perform assay allowing the timely identification of the most common non-typhoidal serovars (NTS) is needed. In this study, a molecular assay based on loop-mediated isothermal amplification (LAMP) targeting specific gene sequences of *Salmonella* Enteritidis, *S.* Typhimurium, *S.* Infantis, *S.* Derby, and *S.* Choleraesuis has been developed for rapid serovar identification from cultured colonies. A total of 318 *Salmonella* strains and 25 isolates of other *Enterobacterales* species that served as negative controls were analyzed. All *S.* Enteritidis (*n* = 40), *S.* Infantis (*n* = 27), and *S.* Choleraesuis (*n* = 11) strains were correctly identified. Seven out of 104 *S.* Typhimurium and 10 out of 38 *S.* Derby strains missed a positive signal. Cross-reactions of the gene targets were only rarely observed and restricted to the *S.* Typhimurium primer set (5 false-positives). Sensitivity and specificity of the assay compared to seroagglutination were as follows: 100% and 100% for *S.* Enteritidis, 93.3% and 97.7% for *S.* Typhimurium, 100% and 100% for *S.* Infantis, 73.7% and 100% for* S*. Derby, and 100% and 100% for *S.* Choleraesuis, respectively. With results available in just a few minutes of hands-on time and a test run time of 20 min, the LAMP assay developed here may be a useful tool for the rapid identification of common *Salmonella* NTS in daily routine diagnostics.

## Introduction

Human salmonellosis is a worldwide public health concern because *Salmonella* spp. is the second leading bacterial pathogen of food-borne infections with the largest numbers of hospitalizations [1–3]. The genus *Salmonella* includes two species, *Salmonella enterica* and *S. bongori*, and is further classified into more than 2600 serovars based on their lipopolysaccharide (LPS) and flagellar antigens according to the White-Kauffmann-Le Minor scheme [4, 5]. Typhoidal serovars (TS, *S.* Typhi, *S.* Sendai, and *S.* Paratyphi A, B, and C) cause systemic infections, known as enteric fever, regardless of the immune status of the patient and are usually acquired through contaminated food and water or by person-to-person transmission [6]. Non-typhoidal serovars (NTS) are zoonotic pathogens that are transmitted to humans following enrichment in contaminated food and cause acute gastroenteritis. Extraintestinal infections and bacteremia can occur in high-risk groups, such as infants, the elderly, or immunocompromised patients [1, 2]. In Sub-Saharan Africa NTS belong to the major causative pathogens of bacteremia in young children and HIV-positive patients [7, 8]. In the European Union (EU), 52,072 cases of salmonellosis with a hospitalization rate of 29.9% were documented in 2020 [9]. More than 60% of them were caused by only five serovars: *S.* Enteritidis, *S.* Typhimurium, the monophasic variant of *S.* Typhimurium, *S.* Infantis, and *S.* Derby [1, 9, 10]. Conventional serotyping is often time-consuming. Whereas the O antigens can immediately be determined by agglutination, the identification of H phases takes some days because phase variation must be induced [5]. Sometimes serotyping results are prone to misinterpretation because of incomplete expression of antigens [11]. Now, whole-genome sequencing (WGS) is being increasingly used not only to identify clonal clusters of isolates during food-borne salmonellosis outbreaks but also to replace antigen agglutination and to determine the serotype by using web-based tools such as SeqSero [5, 12, 13]. These expensive and laborious techniques that require extensive computing are of particular importance for epidemiological surveillance and the work of public health services, but for daily routine diagnostics, an interest in simple and rapid methods to identify common serotypes exists [10, 14]. PCR and loop-mediated isothermal amplification (LAMP) assays targeting genes that are specific for defined serovars are an option to replace initial serotyping and WGS can be used in addition if necessary [11, 15, 16]. LAMP, firstly developed by a Japanese research group in 2000, is characterized by high-speed amplification at constant temperature that relies on an auto-cycling catalyzed by a *Bst* DNA polymerase with strand displacement activity [17]. The specificity of LAMP is generally high because of the use of 4–6 primers for each gene target. First LAMP assays to identify *Salmonella* spp. and a few serovars in clinical and food samples have been described in recent years [10, 18–20].

This study aimed to develop a LAMP-based assay (SalmoTyper) to rapidly identify the most common serotypes *S.* Enteritidis, *S.* Typhimurium including its monophasic variant, *S.* Infantis, and *S.* Derby from isolated bacterial colonies. Additionally, *S.* Choleraesuis, which is a pathogen of concern in our region, was included [21]. The assay has been designed as a user-friendly rapid test with lyophilized master mixes that can be stored at room temperature and do not need any further pipetting steps for preparation.

## Materials and methods

### LAMP primer design and comparative genome in silico analysis

Whole-genome sequences of *Salmonella* serovars were obtained from the National Center for Biotechnology Information (NCBI) database GenBank (https://www.ncbi.nlm.nih.gov/, Bethesda, MD, USA) and compared using the Basic Local Alignment Search Tool (BLAST, https://blast.ncbi.nlm.nih.gov/Blast.cgi) to identify potential serovar-specific genome regions. Whole genome sequences (in GenBank format) of one representative of each serovar were compared in subsections of 100,000 base pairs in length using BLAST search to identify regions of lower sequence homology to other serovars. These regions were then used to calculate LAMP primer sets and the calculated amplicons were in turn analyzed for specificity by BLAST analysis once again. LAMP primer sets targeting the identified specific DNA sequences were designed via LAMP Designer software 1.16 (PREMIER Biosoft, San Francisco, CA, USA; Table 1).

**Table 1:** Target *Salmonella* genes used for LAMP primers of the SalmoTyper assay

Assay parameter	Gene	Gene product	GenBank accession no	Primer	Sequence (5′-3′)	Amplicon size	Predicted cross-reactions^a^
*Salmonella* spp.	*invA*	Invasion protein InvA	NC_003197.2	F3-SeinvA B3-SeinvA LF-SeinvA LB-SeinvA FIP-SeinvA BIP-SeinvA	ATCGCACTGAATATCGTACTG CCACGGTGACAATAGAGAAG GGTAATTAACAGTACCGCAGGA CTTGATTGAAGCCGATGCC AATGCCAGACGAAAGAGCGTTCGTTCTACATTGACAGAATCC TCGATCAGTACCAGCCGTCTTACGCCAATAACGAATTGCC	163 bp	None
*S.* Enteritidis	C0Z09_12860	Transcriptional regulator	CP025554.1	F3-Salend1 B3-Salend1 LF-Salend1 LB-Salend1 FIP-Salend1 BIP-Salend1	CTGGTACTTACGATGACAACTT GGGAGGGAGGAGCTTTAG TTCTTTCTCAGATTCAGGGAGT CTGCACAAAAGCGCCTAAA ACCTTTAAGCCGGTCAATGAGTCAGACAACAGGCTGATTTACTA AGAGGCTTTCCAGAACATGCTCATGTGGTTGGTTCGTCAC	165 bp	*S.* Javiana *S.* Newlands
*S.* Typhimurium	JYM79_16920	RHS repeat protein	CP074209.1	F3-Stymur1 B3-Stymur1 LF-Stymur1 LB-Stymur1 FIP-Stymur1 BIP-Stymur1	GGCGGGTTAAACCTTTATCA ATGTTTGCTCACCCACAAT CTACTGTCCCAGTATCAAGCAT TTCTATCGAAGGGCGATTAGC GGTAGGCAACATGAGTTGTCCCCTGCGGAATATCAGCAA CCTACAAATGAAGGATGGGCAATGTTGGCATTTCAGGAACA	255 bp	*S.* Abony *S*. Adjame *S.* Thompson *S.* Bredeney *S*. Give *S.* Quebec
*S.* Infantis	yecD_1	Putative isochorismatase hydrolase	LS483479.1	F3-Salinf2 B3-Salinf2 LF-Salinf2 LB-Salinf2 FIP-Salinf2 BIP-Salinf2	GGCTATTCATCCTGATGTCG TATGCCCAGGCTTCTGATA ACACCGGTAAGGATCAGGT CGTTGTGAGTGATTGCTGC AAGCACAACACCACTGGTAGTCAACTTGAGATGATCCTTCGTG TGACCTGGATTACCGCCTCAACATTGGTGTCATGATCTGG	163 bp	*S.* Sloterdijk
*S.* Derby	JF569_ 15,965	YadA-like family protein	CP066545.1	F3-Salder3 B3-Salder3 LF-Salder3 LB-Salder3 FIP-Salder3 BIP-Salder3	AATAGTCAGCCAGTGAATCAC ACCAGTATCACCAACCTGA CATCTACCAGTACCGACGC CATCCCATTTCAGTGCATCTTC CGTCACCGCTGGAACAATCTAAGAGTTGTCCACCGTTAAC CAGCGGTGAATACGCCTGATACGGTGACTAACCTTGGT	159 bp	*S.* Ouakam *S*. California
*S.* Choleraesuis	SCH_ 1198	Predicted antirepressor	CP012344.2	F3-Salchol B3-Salchol LF-Salchol LB-Salchol FIP-Salchol BIP-Salchol	TTGAATTATGGTGGCTTGGGTA GTTACATCGGTGTATTTGCTGAG ATGGTAGTCATAACGACAGCC TTATTTCCATAAGCCACATAAAGACG CATCAATAACGCAAAGTTCAGGTTGATAGCAGCTTGTGGTGATAAGT TTACTTCCTCACTGGCTGTTGCTTCGACAGAACAATCCAGGC	193 bp	None

^a^Amplicon sequence homology > 95%

### Bacterial strains

A total of 316 strains of *Salmonella enterica* and 1 strain of *S. bongori* from the collections of the Institute of Medical Microbiology, Jena, and the Robert Koch Institute, Wernigerode, Germany, were used as samples for the evaluation of the SalmoTyper assay. Twenty-five patient isolates of other *Enterobacterales* species collected at the Institute of Medical Microbiology, Jena, served as negative control strains. All strains were streaked onto Columbia sheep blood agar and Hektoen enteric agar (Oxoid, Thermo Fisher Scientific, Wesel, Germany) and incubated overnight before LAMP testing.

Species were identified using MALDI-TOF (Vitek MS, bioMeriéux, Nürtingen, Germany) and the identity of *Salmonella* strains was confirmed by serotyping using group-specific and monospecific antisera (Sifin Diagnostics, Berlin, Germany) according to the White-Kauffmann-Le Minor scheme.

### SalmoTyper LAMP assay

**Fig. 1:**
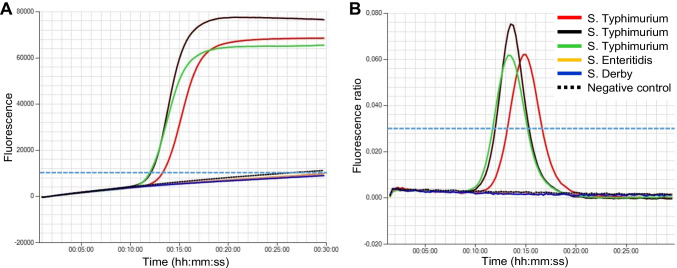
Pre-evaluation of the JYM79_16920 primer set for identification of *S.* Typhimurium using three different strains. S*.* Enteritidis, and *S.* Derby served as controls. The thresholds of the fluorescence level (**a**) and amplification rate (**b**) are marked with a broken light blue line. The fluorescence signal must raise above both thresholds

Each LAMP test strip contained lyophilized master mixes with one specific primer set in each cap and an additional cap containing the inhibition control. The strips were manufactured by the Amplex Diagnostics’ lyophilization service (https://www.eazyplex.com/en-gb/lyophilisierungsservice), using isothermal master mix ISO-004 (Optigene Ltd., Horsham, UK) as basis reagent. A single small colony of the isolates was suspended in 500 μl of resuspension and lysis fluid (RALF buffer, Amplex Diagnostics) and boiled for 2 min. After centrifugation at 4000 rpm for 1 min, 25 μl of the supernatant was added to each tube of the SalmoTyper test strip. Tests were run on a Genie HT machine (Amplex Diagnostics) at 65 °C for 20 min. Amplification was measured by real-time fluorescence detection using a DNA intercalating dye. Data interpretation was automatically performed by the integrated eazyReport™ software (Amplex Diagnostics). Results were reported as positive in real-time if the fluorescence level and the peak of the first derivative of the fluorescence curve had risen above the thresholds of 10,000 and 0.025, respectively. The thresholds represent the standard settings recommended by the manufacturer of the Genie HT instrument. A representative example is shown in Fig. 1.

## Results and discussion

### Evaluation using characterized *Salmonella* isolates

For the evaluation of the assay 307 isolates of *S. enterica ssp. enterica* covering 43 serovars were used (Table 2). Representative strains of the following subspecies and species were additionally examined: *S. enterica* ssp. *salamae* (*n* = 3), *S. enterica* ssp. *arizonae* (*n* = 4), *S. enterica* ssp. *diarizonae* (*n* = 1), *S. enterica* ssp. *houtenae* (*n* = 1), and *S. bongori* (*n* = 1).

**Table 2:** Serovars of *S. enterica* spp. *enterica* included in this study

Group	Serovar	Antigenic formula	*n*
A	*S.* Paratyphi A	1,2,12:a [1, 5]:	4
B	***S.* Typhimurium**	1,4,[5],12:i:1,2	86
B	***S.* Typhimurium, monophasic**	1,4,[5],12:i: –	18
B	***S.* Derby**	1,4,[5],12:f,g [1, 2]:	38
B	*S.* Abony	1,4,[5],12,[27]: b: e,n,x	6
B	*S.* Agona	1,4,[5],12:f,g,s [1, 2]:	2
B	*S.* Brandenburg	4,[5],12:l,v:e,n,z15	5
B	*S.* Bredeney	1,4,12,27:l,v:1,7	1
B	*S.* Paratyphi B	1,4,[5],12:b:1,2	6
B	*S.* Reading	1,4,[5],12:e,h:1,5	1
B	*S.* Saintpaul	1,4,[5],12:e,h:1,2	2
B	*S.* Stanley	1,4,[5],12,[27]:d:1,2	1
B	*S.* Coeln	1,4,[5],12:y:1,2	1
C1	***S.* Infantis**	6,7,14:r:1,5	27
C1	***S.* Choleraesuis**	6,7:c:1,5	11
C1	*S.* Braenderup	6,7,14:e,h:e,n,z15	1
C1	*S.* Livingstone	6,7,14:d:l,w	1
C1	*S.* Mbandaka	6,7,14:z10:e,n,z15	2
C1	*S.* Oranienburg	6,7,14:m,t:[z57]	1
C1	*S.* Paratyphi C	6,7,[Vi]:c:1,5	4
C1	*S.* Rissen	6,7,14:f,g:–	3
C1	*S.* Thompson	6,7,14:k:1,5	2
C1	*S.* Virchow	6,7,14:r:1,2	3
C1	*S.* Tennessee	6,7,14:z29 [1, 2, 7]:	1
C2-C3	*S.* Bonariensis	6,8:i:e,n,x	1
C2-C3	*S.* Bovismorbificans	6,8,20:r,[i]:1,5	2
C2-C3	*S.* Goldcoast	6,8:r:l,w	8
C2-C3	*S.* Hadar	6,8:z10:e,n,x	2
C2-C3	*S.* Kentucky	8,20:i:z6	2
C2-C3	*S.* Kottbus	6,8:e,h:1,5	1
C2-C3	*S.* Manhattan	6,8:d:1,5	1
C2-C3	*S.* Newport	6,8,20:e,h:1,2	4
C2-C3	*S.* Stourbridge	6,8:b:1,6	1
D1	***S.* Enteritidis**	1,9,12:g,m:–	40
D1	*S.* Javiana	1,9,12:l,z28:1,5	1
D1	*S.* Napoli	1,9,12:l,z13:e,n,x	1
D1	*S.* Panama	1,9,12:l,v:1,5	2
D1	*S.* Typhi	9,12[Vi]:d:–	7
E1	*S.* Anatum	3,10:e,h:1,6	1
E1	*S.* Give	3,{10}{15}{15,34}:l,v:1,7	1
E1	*S.* London	3,{10}{15}:l,v:1,6	2
E1	*S.* Weltevreden	3,{10}{15}:r:z6	2
G	*S.* Adjame	13,23:r:1,6	1

Targets of the SalmoTyper LAMP assay are printed in bold

The serovar-specific primers were selected for the criterium of exclusivity based on the in silico comparative genome analysis, but their target genes do not necessarily represent specific virulence genes or O and H antigen associated markers. The assay also includes a primer set for the type III secretion system-associated *invA*, a widely used gene target to confirm the genus of *Salmonella* [13, 18]. All strains included in the study signaled positive using the selected *invA* primers (Table 3).

**Table 3:** Identification of *S.* Typhimurium, *S.* Enteritidis, *S.* Derby, *S.* Infantis, and *S.* Choleraesuis strains by the LAMP SalmoTyper assay

Serotype	*n*	Number of positive LAMP results, mean values of threshold time (SD^a^) in min						
		*Salmonella* spp.	*S.* Typhimurium	*S.* Enteritidis	*S.* Derby	*S.* Infantis	*S.* Choleraesuis	IC^b^
*S.* Enteritidis	40	40, 4.5 (0.75)	0	40, 5.5 (0.75)	0	0	0	40, 9.25 (0.75)
*S.* Typhimurium^c^	104	104, 5.25 (0.5)	97, 10.75 (1)	0	0	0	0	104, 9 (0.75)
*S.* Infantis	27	27, 5.25 (0.5)	0	0	0	27, 5.25 (0.5)	0	27, 9 (0.75)
*S.* Derby	38	38, 5 (0.5)	0	0	28, 5.5 (0,75)	0	0	38, 9 (0.75)
*S.* Choleraesuis	11	11, 4.5 (0.25)	0	0	0	0	11, 5.25 (0.25)	11, 9 (0.5)
Other NTS and TS isolates	97	97, 5.25 (1)	5, 10.25 (0.5)^d^	0	0	0	0	97, 8.75 (0.5)

^a^*SD* standard deviation

^b^*IC* inhibition control

^c^Including isolates of the monophasic variant

^d^*S.* Abon*y* (*n* = 2), *S*. Adjame (*n* = 1), *S.* Thompson (*n* = 2)

All *S.* Enteritidis strains (*n* = 40) were correctly identified (Table 3). *S.* Javiana, a serovar with an in silico predicted cross-reaction for the primer set, did not give a positive signal (Table 1). *S.* Enteritidis was discovered by August Gärtner, first chair of hygiene at the University of Jena, during an epidemic of meat poisoning in 1888 [22]. In 2020, it was the most frequent NTS in humans in the EU [9]. Although *S.* Enteritidis is distributed among different animals; it is mainly associated with poultry and survives in the reproductive tissue and the environment of the egg. A recent multi-country outbreak by *S.* Enteritidis in the EU was recorded from September 2021 till January 2022 and traced back to eggs originated from three farms [23]. The strain of ST11 responsible for this outbreak is suggested to be widely distributed in the food production chain.

*S.* Typhimurium, the most prevalent NTS worldwide and on second rank in the EU, is also associated with several animal species, and it is the most frequent serovar isolated from beef [1, 24]. In our study, 97 out of 104 isolates of *S.* Typhimurium could be identified with the selected primer set (Table 3). In silico predicted cross-reactions of the primers were confirmed for *S.* Abony, *S.* Adjame, and *S.* Thompson, but not observed for *S.* Bredeney and *S.* Give (Tables 1 and 3). The LAMP assay cannot differentiate between *S.* Typhimurium and its monophasic variant, but sometimes, it may be important to identify it by seroagglutination or WGS [8, 24, 25]. In 2022, a multi-country salmonellosis outbreak caused by contaminated chocolate products was attributed to the monophasic variant of *S.* Typhimurium of ST34 with buttermilk as a possible contamination source [26]. It is of note that there are strains of this variant that can utilize lactose and may be overlooked on common selective agars [25].

All *S.* Infantis strains (*n* = 27) were detected by the primer set and cross-reactions with other serovars included in the study were not observed (Table 3). *S.* Infantis is the third most prevalent serovar in the EU and mainly associated with poultry sources [9, 27–29]. In the EU, the number of cases significantly increased between 2011 and 2013 due to the spread of several clones from broilers including a dominant Hungarian clone [27]. A large outbreak in humans occurred in the federal state of Thuringia, Germany, and regions of neighboring federal states in 2013 [30]. Two hundred sixty-seven cases were notified and all of them could be traced back to one slaughterhouse. Strains associated with such outbreaks can circulate for more than 10 years in the food chain [29]. The environmental persistence of *S.* Infantis may be explained by the production of biofilms on stainless-steel surfaces [30].

*S.* Derby has ranked among the most frequent serovars since 2006. It is the most prevalent NTS isolated from pork meat [31, 32]. The specific LAMP primer set targeting a region in the Yad-like family adhesion protein missed 10 out of 38 isolates (Table 3). These isolates belonged to the MLST sequence types ST632 and ST71 [33]. *S.* Derby is considered as a serotype of polyphyletic nature [31]. ST632 has caused an outbreak in 2013 and 2014 in Berlin and the federal state of Brandenburg, Germany, with raw pork sausages identified as the source of infection [33]. ST71 is a rarely isolated sequence type commonly distributed in poultry [31, 34]. The other isolates belonged to the most frequent serotypes ST39 and ST40 and were identified by the LAMP assay [32–34].

All strains of *S.* Choleraesuis (*n* = 11) were detected and there were no positive signals by other serovars (Table 3). Concerning the number of infections *S.* Choleraesuis possesses a higher invasive index than other NTS. Whereas less than 2% of *S.* Enteritidis and *S.* Typhimurium cases are associated with bacteremia and extraintestinal manifestations, this takes place in more than 50% of *S.* Choleraesuis infections [6]. *S.* Choleraesuis is present in pigs and known as the causative agent of swine edema, a severe systemic infection [23]. It is genetically related to *S.* Paratyphi C with which it shares the same antigenic formula. Because of its increased virulence, this serovar has been included in the assay despite a rather low prevalence.

### Performance of the SalmoTyper assay

Overall, the LAMP assay showed an acceptable accuracy in comparison to serovar agglutination (Table 4). *S.* Enteritidis, *S.* Infantis, and *S.* Cholerasuis were identified with 100% sensitivity and specificity. For *S.* Typhimurium, both parameters were lower, but test performance reached a Cohen’s *κ* value of 0.91. A limitation of the assay has to be considered for the identification *S.* Derby because of a sensitivity of only 73.7% (Table 4). A great advantage of the LAMP technology is the fast time to result. Sample preparation took only a few minutes, and the mean threshold time for fluorescence intensity values of specific targets ranged from 4.5 to 10.25 min. IC was detected at a mean time of 9 min (Table 3).

**Table 4:** Performance data of the SalmoTyper assay

LAMP target	True-positive (n)	True-negative (n)	False-positive (n)	False-negative (n)	Sensitivity, % (CI^a^)	Specificity, % (CI^a^)	Cohen’s *K* (CI^c^)	Scale
*S.* Enteritidis	40	277	0	0	100 (91.2–100)	100 (98.7–100)	1	Almost perfect agreement
*S.* Typhimurium^d^	97	208	5	7	93.3 (86.6–97.2)	97.7 (94.6–99.2)	0.91 (0.87–0.96)	Almost perfect agreement
*S.* Infantis	27	290	0	0	100 (87.2–100)	100 (98.7–100)	1	Almost perfect agreement
*S.* Derby	28	279	0	10	73.7 (56.9–86.6)	100 (98.7–100)	0.83 (0.73–0.93)	Almost perfect agreement
*S.* Choleraesuis	11	306	0	0	100 (71.5–100)	100 (98.8–100)	1	Almost perfect agreement
*Salmonella* spp.^c^	317	25	0	0	100 (98.5–100)	100 (86.3–100)	1	Almost perfect agreement

^a^*CI* 95% confidence interval

^b^Including isolates of the monophasic variant

^c^Evaluation of the *Salmonella* spp. target included 25 strains of other *Enterobacterales* species

To exclude that the primer sets may cross-react with other *Enterobacterales* species, 25 clinical isolates with a similar growth characteristics compared to *Salmonella* on common selective agars such as Hektoen enteric and *Salmonella Shigella* agar (H_2_S-positive and/or lactose-negative) were examined. Negative control strains included *Citrobacter freundii* complex (*n* = 3), *C. koseri* (*n* = 1), lactose-negative *Escherichia coli* (*n* = 5) lactose-negative *Enterobacter cloacae* complex (*n* = 4), *Morganella morganii* (*n* = 1), *Proteus hauseri* (*n* = 1), *P. mirabilis* (*n* = 2), *P. vulgaris* (*n* = 2), *Providencia alcalifaciens* (*n* = 1), *Serratia marcescens* (*n* = 1), *Shigella flexneri* (*n* = 1), *S. sonnei* (*n* = 1), and *Yersinia enterocolitica* (*n* = 2). No isolate showed a positive amplification signal for the *Salmonella*-specific primer sets.

## Conclusions

In recent years, an increasing number of molecular assays have been introduced for identification of salmonellae in clinical and food samples. Most of these assays target the genus of *Salmonella* for culture-independent diagnostic screening of samples, but there are also some test systems allowing the detection of single serovars. The results of this work demonstrate that a LAMP-based assay that uses lyophilized reagents and does not need DNA extraction provides an easy-to-perform diagnostic tool for rapid identification of frequent *Salmonella* serovars. Although there were just a few in silico predictions for cross-reactions for the primer sets defined as serovar-specific, a limitation of this study is the absolute number of serovars that could be included for evaluation.

Some strains of *S.* Typhimurium and *S.* Derby were not detected by the selected primers. However, this has little impact on the usability of the assay for routine diagnostics. Since the majority of isolates can be reliably identified, the workload is reduced and thus processing time is shortened. It should be considered that a risk to overlook distinct clonal clusters within one serovar cannot be fully excluded when mutations in the target regions of the primers occur because the genes that had been chosen for a molecular assay-based identification are not directly linked to synthesis pathways of O and H antigens. Moreover, one should be aware that the local distribution of serovars may vary, and normally, less frequent serovars can change the epidemiological situation following a large outbreak when they persist and circulate for a prolonged time in the food market, such as *S.* Braenderup during an EU multi-country outbreak in 2021 [35]. WGS is the method of choice to identify outbreaks by distinct clones and their sources of infection. The LAMP assay presented here has been designed for implementation as a rapid diagnostic tool to identify frequent NTS with little workload in daily routine diagnostics.

## Data Availability

The dataset analyzed in this study is available from the corresponding author on reasonable request.
